# Higher Delta variant-specific neutralizing antibodies prevented infection in close contacts vaccinated with ancestral mRNA vaccines during the SARS-CoV-2 Delta wave

**DOI:** 10.1038/s41598-023-46800-x

**Published:** 2023-11-07

**Authors:** Yun Shan Goh, Siew-Wai Fong, Matthew Zirui Tay, Angeline Rouers, Zi Wei Chang, Jean-Marc Chavatte, Pei Xiang Hor, Chiew Yee Loh, Yuling Huang, Yong Jie Tan, Bei Wang, Eve Zi Xian Ngoh, Siti Nazihah Mohd Salleh, Raphael Tze Chuen Lee, Georgina Lim, NCID Study Group, Jocelyn Jin Yu, Zheng Kuang Soh, Yi Qing Chin, Jonathan Jordon Lim, Juwinda Ongko, Eshele Anak Libau, Mohammed Ridzwan Bin Abdullah, Shiau Hui Diong, Jefanie Teo, He Ping Yeo, COVID-Study Group, Adeline C. Y. Chua, Anthony Torres-Ruesta, Siti Naqiah Amrun, Nicholas Kim-Wah Yeo, Vanessa Kexin Neo, Wendy Yehui Chen, Isaac Kai Jie Kam, Alice Soh Meoy Ong, Estelle Yi Wei Goh, Nathan Wong, Zhi Feng Sherman Lim, Sebastian Maurer-Stroh, Cheng-I Wang, Yee‐Sin Leo, Raymond T. P. Lin, Meng Chon Lam, David C. Lye, Barnaby Edward Young, Lisa F. P. Ng, Laurent Renia

**Affiliations:** 1 A*STAR Infectious Diseases Labs (A*STAR ID Labs), Agency for Science, Technology and Research (A*STAR), 8A Biomedical Grove, Immunos #05-13, Singapore, 138648 Singapore; 2 National Public Health Laboratory, National Centre for Infectious Diseases, Singapore, Singapore; 3 Singapore Immunology Network, A*STAR, Singapore, Singapore; 4 Bioinformatics Institute, A*STAR, Singapore, Singapore; 5 GISAID Global Data Science Initiative (GISAID), Munich, Germany; 6 Ministry of Health (MOH), Singapore, Singapore; 7 National Centre for Infectious Diseases (NCID), Singapore, Singapore; 8 Department of Medicine, Yong Loo Lin School of Medicine, National University of Singapore, Singapore, Singapore; 9 Department of Biological Sciences, National University of Singapore, Singapore, Singapore; 10 Lee Kong Chian School of Medicine, Nanyang Technological University, Singapore, Singapore; 11 Department of Infectious Diseases, Tan Tock Seng Hospital, Singapore, Singapore; 12 Saw Swee Hock School of Public Health, National University of Singapore, Singapore, Singapore; 13 Department of Microbiology and Immunology, Yong Loo Lin School of Medicine, National University of Singapore, Singapore, Singapore; 14 Health Protection Research Unit in Emerging and Zoonotic Infections, National Institute of Health Research, University of Liverpool, Liverpool, UK; 15 Institute of Infection, Veterinary and Ecological Sciences, University of Liverpool, Liverpool, UK; 16 School of Biological Sciences, Nanyang Technological University, Singapore, Singapore

**Keywords:** Immunology, Diseases, Medical research

## Abstract

Identification of the risk factors and the high-risk groups which are most vulnerable is critical in COVID-19 disease management at a population level. Evaluating the efficacy of vaccination against infections is necessary to determine booster vaccination strategies for better protection in high-risk groups. In this study, we recruited 158 mRNA-vaccinated individuals during the Delta wave of SARS-CoV-2 infections in Singapore and examined the antibody profiles of infected individuals. We found that, despite high exposure due to communal living conditions in proximity, 4% of individuals (6/158) had PCR-confirmed infections and 96% (152/158) remained uninfected. Time-course analysis of the antibody profile at the start and the end of quarantine period showed Delta-specific boosting of anti-spike antibody response in 57% of the uninfected individuals (86/152). In the remaining 43% of the uninfected individuals (66/152) with no Delta-specific antibody boost, we found a higher Delta-specific antibody response at the start of quarantine period, which correlated with higher Delta pseudovirus neutralizing capacity. Our findings indicate that a higher basal variant-specific antibody response in the mRNA-vaccinated individuals contributes to better protection against infections by the new emerging SARS-CoV-2 variants.

## Introduction

COVID-19 vaccines have been essential in bringing the pandemic under control. They have shown to be highly efficacious against severe diseases^1,2^. However, breakthrough infections do occur. Exposure to infected individuals is a major risk factor^3^. Hence, many studies on breakthrough infections are in healthcare settings^4–6^, where the workers are a high-risk group. In Singapore, workers living in dormitories have been identified as a high-risk group due to communal living conditions in proximity within the dormitories^7^. In this high-risk group, exposure to infected cases is high and over an extended period of time. Evaluating the efficacy of vaccination against infections is necessary to determine booster vaccination strategies for better protection in this high-risk group. In this study, we studied the spike-specific antibody profile of two infected individuals and their 156 close contacts and investigated the potential contributory role of antibody in protection against infection.

## Results

### mRNA-vaccinated close contacts of infected cases remained largely uninfected following exposure

**Figure 1:**
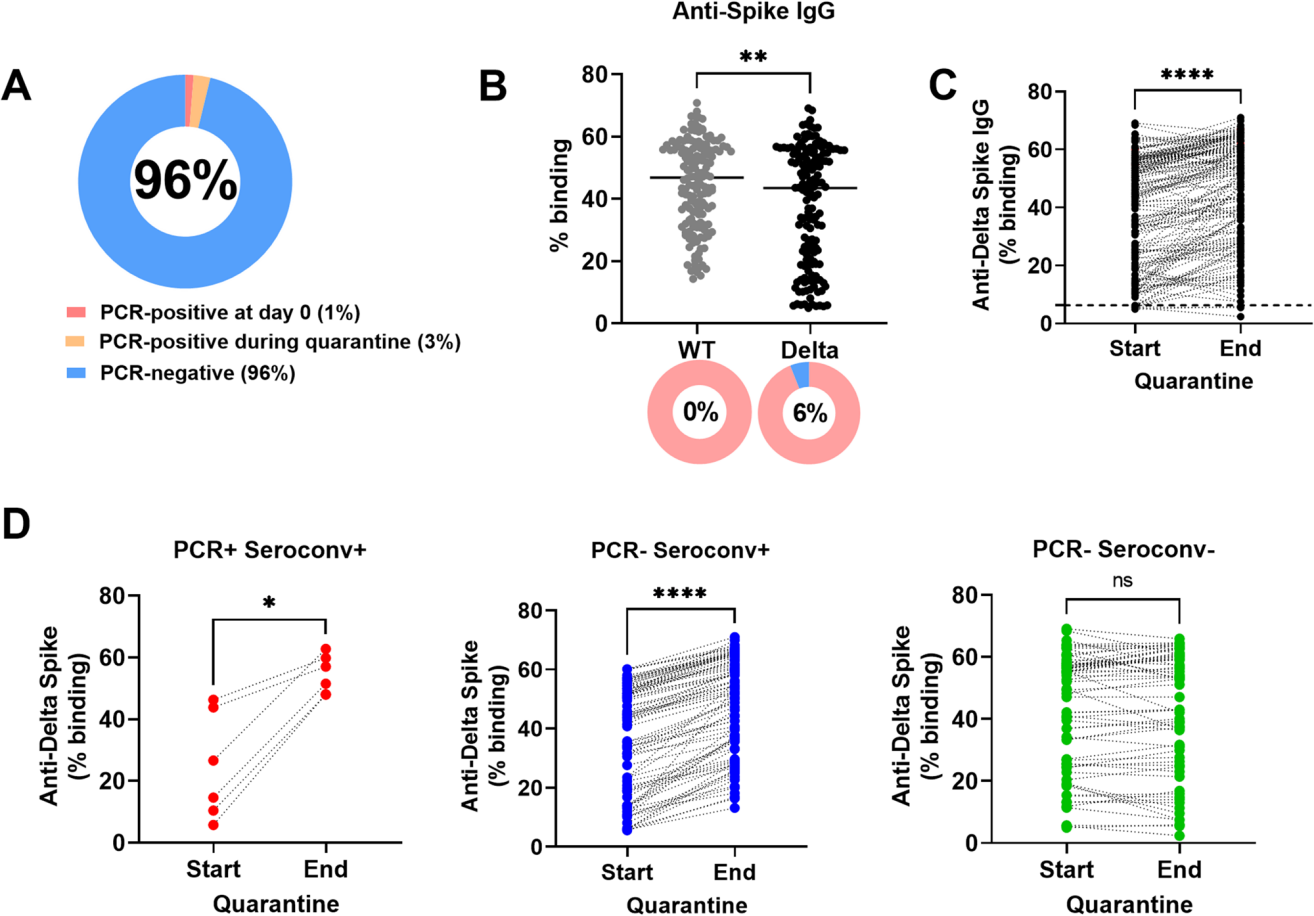
Antibody response against WT and Delta Spike. (**A**) PCR status of cohort (n = 158). (**B**) Plasma samples of all 158 individuals collected at the start of the quarantine (n = 158) were screened for binding by SFB assay. IgG binding against full-length WT and Delta Spike were compared for all 158 individuals (n = 158). Line indicates median IgG response. Pie chart indicates the proportion with positive antibody response (in pink) and proportion with negative response (in blue). Number in pie chart indicates the proportion with negative response. Positive antibody response is defined as response above mean + 3SD of 22 pre-COVID-19 unvaccinated healthy controls^8^. (**C**) Paired analysis of all 158 individuals at the start and end of the quarantine were performed for IgG binding against full-length Delta Spike. Dotted line indicates positive antibody response, defined as response above mean + 3SD of 22 pre-COVID-19 unvaccinated healthy controls^8^. Start, start of quarantine; End, end of quarantine. (**D**) The paired IgG responses at the start and the end of the quarantine of the 158 individuals in C were re-plotted, where the data points were stratified into three groups by PCR status and Delta Spike IgG seroconversion at the end of the quarantine period: (1) PCR-positive and Delta Spike IgG-seroconverted, PCR + Seroconv + (n = 6), (2) PCR-negative and Delta Spike IgG-seroconverted, PCR- Seroconv + (n = 86) and (3) PCR-negative without Delta Spike IgG-seroconversion, PCR- Seroconv- (n = 66). The IgG responses at start and end of the quarantine (from **C**) for the three groups were re-plotted. Start, start of quarantine; End, end of quarantine. Delta Spike IgG-seroconversion was defined as having an increase of Delta-Spike IgG binding of > 6.3% (mean + 3SD of 22 pre-COVID-19 unvaccinated healthy controls). To compare between two groups, Mann Whitney U-tests were used. For paired analysis, Wilcoxon tests were used. *p < 0.05; **p < 0.01; ****p < 0.0001; ns, non-significant.

Workers living in dormitories have been identified as a high-risk group in Singapore due to communal living conditions in proximity within the dormitories^7^. As part of routine surveillance, we identified two individuals with confirmed infection (by PCR) and their 156 close contacts during the Delta wave of SARS-CoV-2 infections in Singapore. Upon identification of the two PCR-positive individuals, we recruited all 158 individuals into a prospective study and followed them for a 14-days quarantine period. All individuals were mRNA-vaccinated (Table 1). Blood samples were collected as soon as possible after confirmation of infection by PCR. Blood samples were also collected from the 156 close contacts at the start of the study. A second blood sample was collected from all 158 individuals at the end of the quarantine. Out of these 158 individuals (Fig. 1A), 2/158 (1%) were PCR-positive at the start of quarantine. Both individuals were isolated and received full medical treatment immediately. By the end of the quarantine follow-up period, an additional 4/158 (3%), who were initially PCR-negative at the start of quarantine, were found to be PCR-positive Delta cases and were isolated for full medical treatment immediately. All PCR-positive cases were confirmed as Delta cases by direct sequencing. As a result, a total of 152/158 (96%) individuals remained PCR-negative at the end of quarantine.

**Table 1:** Demographic information of study cohort.

	N = 158
Age, median (range), years	34 (23–54)
Gender, n (%)	
Male	158 (100)
Female	0 (0)
Ethnicity, n (%)	
Chinese	0 (0)
Indians	153 (96.8)
Malays	0 (0)
Others	5 (3.2)
Prior infection, n (%)	
With prior infection	52 (32.9)
With no prior infection	106 (67.1)

### Variant spike-specific antibody response was rapidly induced following exposure

Using a flow cytometry-based assay (SFB) that measures antibody response against full-length Spike as a marker of previous infection or exposure^8^, we found antibody response against WT Spike in all 158 individuals at the start of quarantine (Fig. 1B). However, the antibody response against Delta Spike was significantly lower than the antibody response against WT Spike (Fig. 1B). A total of 9/158 individuals (6%) did not have antibodies against Delta Spike. Using the Delta SFB assay, we observed a statistically significant difference in antibodies against Delta Spike between the start and the end of quarantine (Fig. 1C). Out of the 152 uninfected individuals, Delta-specific boosting of anti-spike antibody response was observed in 57% of the individuals (86/152) while there was no Delta-specific antibody boost in the remaining 43% of the individuals (66/152). We then stratified all 158 individuals into three groups by PCR status and Delta Spike IgG seroconversion at the end of the quarantine period (Fig. 1D): (1) PCR-positive and Delta Spike IgG-seroconverted (n = 6), (2) PCR-negative and Delta Spike IgG-seroconverted (n = 86) and (3) PCR-negative without Delta Spike IgG-seroconversion (n = 66). Delta Spike IgG-seroconversion was defined as having an increase of Delta-Spike IgG binding of > 6.3% (mean + 3SD of 22 pre-COVID-19 unvaccinated healthy controls)^8^. The Delta Spike-IgG response were significantly higher at the end of the quarantine for the groups with Delta-Spike IgG-seroconversion (Fig. 1D).

### Higher basal variant spike-specific neutralizing antibody response found in uninfected close contacts

**Figure 2:**
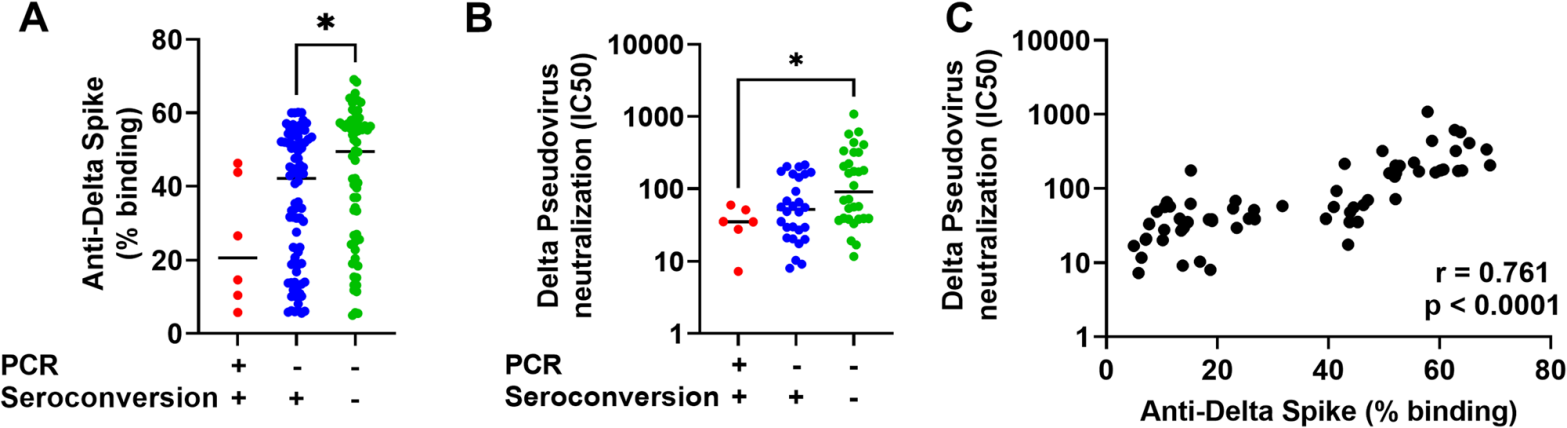
Higher Delta-specific antibody in uninfected vaccinated individuals. Plasma samples were screened for (**A**) IgG binding against full-length Delta and (**B**) Delta pseudovirus neutralization at the start of the quarantine. The samples were classified into three groups: (1) PCR-positive and Delta Spike IgG-seroconverted (n = 6 for both assays), (2) PCR-negative and Delta Spike IgG-seroconverted (n = 86 and n = 30 for Delta-Spike binding and Delta pseudovirus neutralization respectively) and (3) PCR-negative without Delta Spike IgG-seroconversion (n = 66 and n = 29 for Delta-Spike binding and Delta pseudovirus neutralization respectively). To compare between groups at the start of the quarantine, points in A are re-plotted points from Fig. 1D (start of quarantine). Kruskal–Wallis and post hoc tests using Dunn’s multiple comparison tests were used to compare multiple groups. (**C**) Correlation analysis between Delta spike-specific IgG responses and Delta pseudovirus neutralization at the start of the quarantine. Non-parametric Spearman test was used for correlation analysis. *p < 0.05.

We compared the antibody response against Delta Spike at the start of the quarantine between the three groups and found that the proportion of PCR-negative individuals without Delta Spike IgG-seroconversion had a significantly higher baseline antibody response against Delta Spike at the start of the quarantine, compared with PCR-negative individuals with a Delta Spike IgG-seroconversion (Fig. 2A). It is worth noting that the PCR-positive individuals with a Delta Spike IgG-seroconversion group also had a lower baseline antibody response against Delta Spike than PCR-negative individuals with a Delta Spike IgG-seroconversion, though it did not reach statistical significance. The general trend, where the baseline IgG response against Delta Spike at the start of the quarantine is lowest in PCR-positive and Delta Spike IgG-seroconversion-positive group, followed by PCR-negative and Delta Spike IgG-seroconversion-positive group, and lastly PCR-negative and Delta Spike IgG-seroconversion- negative group, was also observed with the Delta pseudovirus neutralization data. PCR-negative individuals without Delta Spike IgG-seroconversion had a significantly higher baseline antibody response at the start of the quarantine, compared with PCR-positive individuals with a Delta Spike IgG-seroconversion (Fig. 2B). We found a significantly strong correlation between anti-Delta Spike antibody binding and Delta pseudovirus neutralization at the start of the quarantine, where the level of anti-Delta Spike antibody positively correlated with the capacity to neutralize Delta pseudovirus (Fig. 2C).

## Discussion

This study highlights the importance of vaccination in individuals who are in a high-risk group due to high exposure. Despite high exposure due to communal living conditions in proximity within the dormitories, 4% of individuals (6/158) had PCR-confirmed infections and 96% (152/158) remained uninfected. Time-course analysis of the antibody profile at the start and the end of quarantine period showed Delta-specific boosting of anti-spike antibody response in 57% of the uninfected individuals (86/152). The sizeable proportion of PCR-negative individuals with a Delta Spike IgG-seroconversion is likely due to high exposure in the dormitories. The rapid induction of antibody response following exposure demonstrates efficient immune priming by vaccination. Interestingly, in the remaining 43% of the uninfected individuals (66/152) with no Delta-specific antibody boost, we found a higher Delta-specific antibody response at the start of quarantine period, which correlated with higher Delta pseudovirus neutralizing capacity. The lack of Delta-specific antibody seroconversion is unlikely due to low exposure as the workers recruited in the study were in proximity with each other. Seroconversion-negative uninfected individuals have been reported in the SARS-CoV-2 human challenge study^9^, despite being inoculated with WT SARS-CoV-2. Similarly, despite being in a high-risk group, a group of healthcare workers has remained seroconversion-negative and uninfected^10^. In the latter study, the authors found stronger, more multi-specific memory T cells against the early transcribed replication–transcription complex in the seroconversion-negative and uninfected individuals, compared with the seroconverted infected individuals. This may have contributed to control of infection. This study presents data from a specific demographic group of male individuals of Indian ethnicity as they are the only groups in large-sized communal living in Singapore. However, it agrees with earlier studies that found association between high neutralizing antibody levels with protection from infection^2,11^. Our findings indicate that a higher basal variant-specific antibody response may also contribute to better protection against infections by the new emerging SARS-CoV-2 variants.

## Methods

### Ethics statement and study population

The study was assessed by Singapore institutional review board, named “National Healthcare Group (NHG) Domain Specific Review Board (DSRB)” (under the purview of the Human Biomedical Research Act, Singapore). The study was approved under the IRB study number 2012/00917, entitled “A Multi-centred Prospective Study to Detect Novel Pathogens and Characterize Emerging Infections (The PROTECT study group). Written informed consent was obtained from all the participants. This is in accordance with Declaration of Helsinki.

As part of surveillance in high-risk groups, we identified two individuals with confirmed Delta infection (by PCR) in the migrant workers’ dormitories during the Delta wave of SARS-CoV-2 infections in Singapore through nasal swab sampling. The migrant workers shared rooms with an occupancy of 12–16 migrant workers. Shared facilities included kitchen, showering and toilet facilities. Prior to the start of the study, there was no restriction in movement within the dormitories. Upon identification, we recruited the two PCR-positive individuals and their 156 close contacts into a prospective study and followed them for a 14-days quarantine period. In addition to the nasal swab samples taken from the individuals at the start of the study, a second nasal swab sample was taken at the end of the quarantine period to identify any additional PCR-positive infections following the quarantine period. All PCR-positive swab samples were confirmed as Delta cases by direct sequencing. All PCR-positive individuals were isolated and received full medical treatment immediately. All individuals were mRNA-vaccinated (Table 1).

A cohort of two infected individuals and 156 close contacts (Table 1), aged 23–54 (median age = 34), were recruited in Aug 2021 during the Delta variant wave. All 156 close contacts live in communal living conditions in proximity to the two infected individuals in the dormitories. All 158 individuals were vaccinated at least 14 days prior to the start of the study, defined in Singapore as either (1) two doses of mRNA primary vaccination (Pfizer/BioNTech BNT162b2 or Moderna mRNA-1273) or (2) one dose of mRNA vaccine after prior SARS-CoV-2 infection. Blood samples were collected as soon as possible after confirmation of infection by PCR. Blood samples were also collected from the 156 close contacts. The time interval between the last vaccine dose and the first blood sample collection ranges from 20 to 199 days (median = 97.5 days). A second blood sample was collected from all 158 individuals at the end of the 14-days quarantine.

### Spike protein flow cytometry-based assay (SFB assay) for antibody detection

The SFB assay was performed as previously described^8,12^. The pTT5LnX-CoV-SP (expressing SARS-CoV-2 Spike protein, Genbank: YP_009724390.1) was used as a template plasmid to generate Spike gene of Delta (B.1.617.2) using QuickChange Lightning MultiSite-Directed Mutagenesis Kit (Agilent)^13^. Cells, expressing spike protein of either WT or Delta, were seeded at 1.5 × 10^5^ cells/well in 96-well V-bottom plates. Cells were incubated with human serum (diluted 1:100 in 10% FBS) followed by a secondary incubation with a double stain, comprising Alexa Fluor 647-conjugated anti-human IgG (1:500 dilution) and propidium iodide (1:2500 dilution). Cells were acquired using BD Biosciences LSR4 and analyzed using FlowJo (Tree Star). Positive antibody response is defined as response above mean + 3SD of 22 pre-COVID-19 unvaccinated healthy controls^8^. The assay was performed as two independent experiments, each with technical duplicates.

### Pseudovirus neutralization assay

Pseudoviruses were generated and tittered as previously described^14^ using a third-generation lentivirus system. The pseudotyped lentivirus neutralization assay was performed as previously described^13–16^, with modifications. A stable cell line expressing human ACE2, CHO-ACE2^17^, was used. Four-fold serially diluted heat‐inactivated samples (1:5 to 1:5120) were incubated with pseudovirus expressing Delta Spike (5 ng p24 per well), before being added to pre‐seeded CHO‐ACE2 cells in duplicate. After 48 h, cells were lysed, and luciferase activity was quantified on GloMax Luminometer (Promega).

### Statistical analysis

Statistical analysis was performed using GraphPad Prism 9. To compare between antibody response against WT and Delta Spike, Mann–Whitney U-test was used. To compare between multiple groups, Kruskal–Wallis tests and post hoc tests using Dunn’s multiple comparison tests were used. For paired analysis, Wilcoxon tests were used. For correlation analysis between anti-Delta Spike antibody and Delta pseudovirus neutralization, spearman correlation was used. *p* < 0.05 was considered statistically significant.

## Data Availability

Data are available from the corresponding author upon request.

## References

[1] Hotez PJ, Nuzhath T, Callaghan T, Colwell B (2021). COVID-19 vaccine decisions: considering the choices and opportunities. Microbes Infect.

[2] McDonald I, Murray SM, Reynolds CJ, Altmann DM, Boyton RJ (2021). Comparative systematic review and meta-analysis of reactogenicity, immunogenicity and efficacy of vaccines against SARS-CoV-2. NPJ Vaccines.

[3] Alishaq M (2021). Risk factors for breakthrough SARS-CoV-2 infection in vaccinated healthcare workers. PLoS One.

[4] Bergwerk M (2021). Covid-19 breakthrough infections in vaccinated health care workers. N Engl J Med.

[5] Rovida F (2021). SARS-CoV-2 vaccine breakthrough infections with the alpha variant are asymptomatic or mildly symptomatic among health care workers. Nat Commun.

[6] Yang SL (2022). COVID-19 breakthrough infections and humoral immune response among BNT162b2 vaccinated healthcare workers in Malaysia. Emerg Microbes Infect.

[7] Gorny AW (2021). SARS-CoV-2 in migrant worker dormitories: Geospatial epidemiology supporting outbreak management. Int J Infect Dis.

[8] Goh YS (2021). Sensitive detection of total anti-Spike antibodies and isotype switching in asymptomatic and symptomatic individuals with COVID-19. Cell Rep Med.

[9] Killingley B (2022). Safety, tolerability and viral kinetics during SARS-CoV-2 human challenge in young adults. Nat Med.

[10] Swadling L (2022). Pre-existing polymerase-specific T cells expand in abortive seronegative SARS-CoV-2. Nature.

[11] Khoury DS (2021). Neutralizing antibody levels are highly predictive of immune protection from symptomatic SARS-CoV-2 infection. Nat Med.

[12] Goh YS, Ng LFP, Renia L (2021). A flow cytometry-based assay for serological detection of anti-spike antibodies in COVID-19 patients. STAR Protoc.

[13] Wang B (2021). Resistance of SARS-CoV-2 variants to neutralization by convalescent plasma from early COVID-19 outbreak in Singapore. NPJ Vaccines.

[14] Poh CM (2020). Two linear epitopes on the SARS-CoV-2 spike protein that elicit neutralising antibodies in COVID-19 patients. Nat Commun.

[15] Renia L (2022). Lower vaccine-acquired immunity in the elderly population following two-dose BNT162b2 vaccination is alleviated by a third vaccine dose. Nat Commun.

[16] Tay MZ (2022). Decreased memory B cell frequencies in COVID-19 delta variant vaccine breakthrough infection. EMBO Mol Med.

[17] Lip KM (2006). Monoclonal antibodies targeting the HR2 domain and the region immediately upstream of the HR2 of the S protein neutralize in vitro infection of severe acute respiratory syndrome coronavirus. J Virol.

