# Author Correction: Higher Delta variant-specific neutralizing antibodies prevented infection in close contacts vaccinated with ancestral mRNA vaccines during the SARS-CoV-2 Delta wave

**DOI:** 10.1038/s41598-024-51484-y

**Published:** 2024-01-17

**Authors:** Yun Shan Goh, Siew-Wai Fong, Matthew Zirui Tay, Angeline Rouers, Zi Wei Chang, Jean-Marc Chavatte, Pei Xiang Hor, Chiew Yee Loh, Yuling Huang, Yong Jie Tan, Bei Wang, Eve Zi Xian Ngoh, Siti Nazihah Mohd Salleh, Raphael Tze Chuen Lee, Georgina Lim, NCID Study Group, Jocelyn Jin Yu, Zheng Kuang Soh, Yi Qing Chin, Jonathan Jordon Lim, Juwinda Ongko, Eshele Anak Libau, Mohammed Ridzwan Bin Abdullah, Shiau Hui Diong, Jefanie Teo, He Ping Yeo, COVID-Study Group, Adeline C. Y. Chua, Anthony Torres-Ruesta, Siti Naqiah Amrun, Nicholas Kim-Wah Yeo, Vanessa Kexin Neo, Wendy Yehui Chen, Isaac Kai Jie Kam, Alice Soh Meoy Ong, Estelle Yi Wei Goh, Nathan Wong, Zhi Feng Sherman Lim, Sebastian Maurer-Stroh, Cheng-I Wang, Yee‐Sin Leo, Raymond T. P. Lin, Meng Chon Lam, David C. Lye, Barnaby Edward Young, Lisa F. P. Ng, Laurent Renia

**Affiliations:** 1 A*STAR Infectious Diseases Labs (A*STAR ID Labs), Agency for Science, Technology and Research (A*STAR), 8A Biomedical Grove, Immunos #05-13, Singapore, 138648 Singapore; 2 National Public Health Laboratory, National Centre for Infectious Diseases, Singapore, Singapore; 3 Singapore Immunology Network, A*STAR, Singapore, Singapore; 4 Bioinformatics Institute, A*STAR, Singapore, Singapore; 5 GISAID Global Data Science Initiative (GISAID), Munich, Germany; 6 Ministry of Health (MOH), Singapore, Singapore; 7 National Centre for Infectious Diseases (NCID), Singapore, Singapore; 8 Department of Medicine, Yong Loo Lin School of Medicine, National University of Singapore, Singapore, Singapore; 9 Department of Biological Sciences, National University of Singapore, Singapore, Singapore; 10 Lee Kong Chian School of Medicine, Nanyang Technological University, Singapore, Singapore; 11 Department of Infectious Diseases, Tan Tock Seng Hospital, Singapore, Singapore; 12 Saw Swee Hock School of Public Health, National University of Singapore, Singapore, Singapore; 13 Department of Microbiology and Immunology, Yong Loo Lin School of Medicine, National University of Singapore, Singapore, Singapore; 14 Health Protection Research Unit in Emerging and Zoonotic Infections, National Institute of Health Research, University of Liverpool, Liverpool, UK; 15 Institute of Infection, Veterinary and Ecological Sciences, University of Liverpool, Liverpool, UK; 16 School of Biological Sciences, Nanyang Technological University, Singapore, Singapore

Correction to: *Scientific Reports* 10.1038/s41598-023-46800-x, Published online 07 November 2023

In the original version of this article, Cheng-I Wang was incorrectly affiliated with ‘National Public Health Laboratory, National Centre for Infectious Diseases, Singapore, Singapore’. The correct affiliation is:

Singapore Immunology Network, A*STAR, Singapore, Singapore

The original article has been corrected.

